# Quantitative mass spectrometric analysis of the mouse cerebral cortex after ischemic stroke

**DOI:** 10.1371/journal.pone.0231978

**Published:** 2020-04-21

**Authors:** Ank Agarwal, Seongje Park, Shinwon Ha, Ji-Sun Kwon, Mohammed Repon Khan, Bong Gu Kang, Ted M. Dawson, Valina L. Dawson, Shaida A. Andrabi, Sung-Ung Kang

**Affiliations:** 1 Neuroregeneration and Stem Cell Programs, Institute for Cell Engineering, Johns Hopkins University School of Medicine, Baltimore, Maryland, United States of America; 2 Department of Chemistry, University of Southern California, Los Angeles, California, United States of America; 3 Department of Neurology, Johns Hopkins University School of Medicine, Baltimore, Maryland, United States of America; 4 Solomon H. Snyder Department of Neuroscience, Johns Hopkins University School of Medicine, Baltimore, Maryland, United States of America; 5 Adrienne Helis Malvin Medical Research Foundation, New Orleans, Louisiana, United States of America; 6 Department of Physiology, Johns Hopkins University School of Medicine, Baltimore, Maryland, United States of America; 7 Department of Pharmacology and Molecular Sciences, Johns Hopkins University School of Medicine, Baltimore, Maryland, United States of America; 8 Department of Pharmacology and Toxicology, and Department of Neurology, University of Alabama at Birmingham, Birmingham, Alabama, United States of America; Chinese Academy of Medical Sciences Institute of Basic Medical Sciences, CHINA

## Abstract

Ischemic strokes result in the death of brain tissue and a wave of downstream effects, often leading to lifelong disabilities or death. However, the underlying mechanisms of ischemic damage and repair systems remain largely unknown. In order to better understand these mechanisms, TMT-isobaric mass tagging and mass spectrometry were conducted on brain cortex extracts from mice subjected to one hour of middle cerebral artery occlusion (MCAO) and after one hour of reperfusion. In total, 2,690 proteins were identified and quantified, out of which 65% of the top 5% of up- and down-regulated proteins were found to be significant (p < 0.05). Network-based gene ontology analysis was then utilized to cluster all identified proteins by protein functional groups and cellular roles. Although three different cellular functions were identified—organelle outer membrane proteins, cytosolic ribosome proteins, and spliceosome complex proteins—several functional domains were found to be common. Of these, organelle outer membrane proteins were downregulated whereas cytosolic ribosome and spliceosome complex proteins were upregulated, indicating that major molecular events post-stroke were translation-associated and subsequent signaling pathways (e.g., poly (ADP-ribose) (PAR) dependent cell death). By approaching stroke analyses via TMT-isobaric mass tagging, the work herein presents a grand scope of protein-based molecular mechanisms involved with ischemic stroke recovery.

## Introduction

In 2013, 6.5 million people died of stroke globally, making it the second-leading cause of death [1]. Pathologically, there are two main types of stroke, hemorrhagic and ischemic; hemorrhagic events account for only 15% of all stroke cases while ischemic strokes account for almost 85% [2]. Whereas hemorrhagic stroke is caused by intracerebral bleeding via vessel rupture, ischemic insult is induced by vessel occlusions that restrict the passage of blood and thus, key nutrients and oxygen to the brain [2]. Ischemic damage pathways are further divided into those that are directly and indirectly due to stroke pathology. All indirect insults are caused by loss of cellular ATP levels, whereas direct insults are due to loss of constant nutrient and oxygen flow [3–5]. Because both sets of mechanisms are poorly understood, many previous stroke studies have focused on identifying elements of stroke etiology.

In the past, other research groups have directed their attempts to high-throughput cDNA microarray and whole-brain mRNA quantification techniques, as well as *in-situ* hybridization for gene expression analysis [3, 6–8]. One study concluded that almost 7% of the genome was regulated at the transcriptional level by a factor of 10 post-ischemic insult [3] while another showed that the level of gene expression regulation differed between the ischemic core, penumbra, and whole brain [7].

However, such investigations have certain downfalls. Genomic analyses cannot differentiate between direct and indirect stroke-induced gene expression changes due to the variety of disease processes [3, 9] and also cannot identify non-transcriptional means of regulation that may affect protein levels [6]. To understand the large-scale changes directly due to stroke mechanisms, it would be more unequivocal to study changes at the translational level.

Although ischemia most dramatically damages the hemisphere directly affected by reduction in blood flow, the contralateral hemisphere is also affected through indirect effects such as changes in protein expression. A previous investigation, which used western blots to analyze murine protein levels, showed that metabolic energy pathways were significantly reduced [3]. Interestingly, more proteins were down-regulated in the directly affected hemisphere whereas more proteins were up-regulated in the contralateral hemisphere [3].

To increase the accuracy of protein expression analysis, other investigations have harnessed real-time PCR for quantification of protein levels. Nevertheless, this method only allows for quantification of specified proteins [10]. In contrast, the first human ischemic stroke project used gel electrophoresis studies to expand the number of quantified proteins and obtain findings more relevant to humans [11]. The team found 132 proteins to be differentially expressed between core and peripheral stroke regions [11]. Of these, only 39 were identified via mass spectrometry and 10 validated via western blot [11]. In order to yield a more expansive and quantifiable group of affected proteins, an unbiased molecular screen was conducted utilizing TMT-isobaric mass tagging. The investigation modeled optimal treatment conditions via measurement of protein levels after ischemia and 1 hour of reperfusion [12].

## Materials and methods

### Middle Cerebral Artery Occlusion (MCAO)

All experimental protocols using animals were approved by the Institutional Animal Care and Use committee of Johns Hopkins University. Middle cerebral artery occlusion (MCAO) was applied only to males in order to avoid sex-dependent molecular variations in cell death. Wild-type littermates were subjected to MCAO with subsequent reperfusion as described previously [11]. In brief, mice were anesthetized with 1.5–2% isoflurane and maintained at normothermic temperature. The occlusion was performed by placing a monofilament to the base of the middle cerebral artery. The procedure was monitored by laser-Doppler flowmetry with a probe placed on a thinned skull over the lateral parietal cortex. After 60 minutes of occlusion, the filament was removed, and the reperfusion was verified. Another 60 minutes later, animal brains were coronally sectioned into five 2-mm-thick sections in a mouse brain matrix and stained in 2% 2,3,5-triphenyltetrazolium chloride solution before fixation in 10% formalin overnight. The infarction area was imaged with a digital camera. After performing MCAO on 6 male mice, half were sacrificed immediately after one hour, and the remaining half were allowed to reperfuse for one hour before being sacrificed. Mice were sacrificed at these pre-determined time points by an overdose with pentobarbital.

### Sample preparation and TMT labeling

The cerebral cortex brain region was collected for each condition. The tissue was treated with lysis buffer (2% SDS, 50 mM triethyl ammonium bicarbonate (TEABC), 5 mM sodium fluoride, 1 mM sodium orthovanadate, and 1 mM β-glycerophosphate). Subsequently, samples were sonicated (20 sec on/off for 10 times at 4°C water bath) and centrifuged (16,000g on 15°C for 20 min). Protein concentration was determined using the Bicinchoninic acid assay (Pierce, Waltham, MA, USA). Cysteine chains from equal amounts of protein (1 mg) from each condition were reduced using 5 mM DTT at 55˚C for 60 min and alkylated using 5 mM iodoacetamide for 45 min at room temperature (RT) in the dark. The samples were then trypsinized for 3 hours for labeling. Tandem mass tag (TMT) labeling was carried out as per the manufacturer instructions with minor modifications. Briefly, trypsinized peptides from two conditions were reconstituted in 50 mM TEABC buffer and mixed with the TMT reagent and incubated at RT for 1 h. After the labeling, all samples were pooled and desalted using Sep-Pak C18 cartridges.

### Mass spectrometry

Peptide fractions were analyzed on an LTQ-Orbitrap Elite mass spectrometer (Thermo Electron, Bremen, Germany) interfaced with Easy-nLC II nanoflow LC system (Thermo Scientific, Odense, Denmark). The pooled TMT-labeled peptides were reconstituted in 0.1% formic acid and loaded onto a trap column (75 μm x 2 cm) packed in-house with Magic C18 AQ (Michrom Bioresources, Inc., Auburn, CA, USA). Peptides were resolved on an analytical column (75 μm x 50 cm) at a flow rate of 300 nL/min using a linear gradient of 10–35% solvent B (0.1% formic acid in 95% acetonitrile) over 90 min. The total run time, including sample loading and column reconditioning, was 120 min. Data-dependent acquisition with full scans in 350–1700 m/z range was carried out using an Orbitrap mass analyzer at a mass resolution of 120,000 at 400 m/z. The fifteen most intense precursor ions from a survey scan were selected for MS/MS fragmentation using higher-energy collisional dissociation (HCD) fragmentation with 32% normalized collision energy and detected at a mass resolution of 30,000 at 400 m/z. Automatic gain control for full MS was set to 1 × 10^6^ for MS and 5 × 10^4^ ions for MS/MS with a maximum ion injection time of 100 ms. Dynamic exclusion was set to 30 sec. and singly charged ions were rejected. Internal calibration was carried out using the lock mass option (m/z 445.1200025) in ambient air.

### Immunoblot assay

Samples were separated by 8–16% SDS-PAGE and transferred to nitrocellulose membrane (0.45 μm). 5% Difco skim milk (BD Bioscience) in PBST (0.05% Tween 20) was incubated for blocking, and the membranes were applied with specific antibodies as described in the previous materials section. After incubation with horseradish peroxidase-conjugated secondary anti-rabbit or anti-mouse IgG (Amersham Bioscience), the antigen-antibody was detected in X-ray film (AGFA) by an ECL method (Thermo Scientific). Primary antibodies used include the following: rabbit anti-Vimentin (ab137321, Abcam), rabbit anti-GFAP (ab7260, Abcam), rabbit anti-FXR2 (#7098, Cell Signaling), rabbit anti-Annexin2 (ab41803, Abcam) and rabbit anti-beta-Actin HRP conjugate (13E5, Cell Signaling). The western blots were quantified using ImageJ and obtained by averaging the data from three independent experiments. Statistical significance was determined by unpaired student’s t-test using GraphPad prism software.

### Data analysis

Gene Set Enrichment Analysis (GSEA) was used to analyze the data for basic bioinformatics. Additionally, Cytoscape v3.3.0 software with the GlueGo plugin (according to the instruction manual), coupled with R analysis for GO networks and pathways, was used to assess enrichment of protein domain databases.

### Statistical analysis

Quantitative data are presented as the mean ± SEM as calculated by R tools. Statistic tests for GO level were assessed by the right-sided hypergeometric test and the Bonferroni step-down for correction.

## Results

### Quantitative proteomic screening for ischemia damage and repair systems

**Fig 1:**
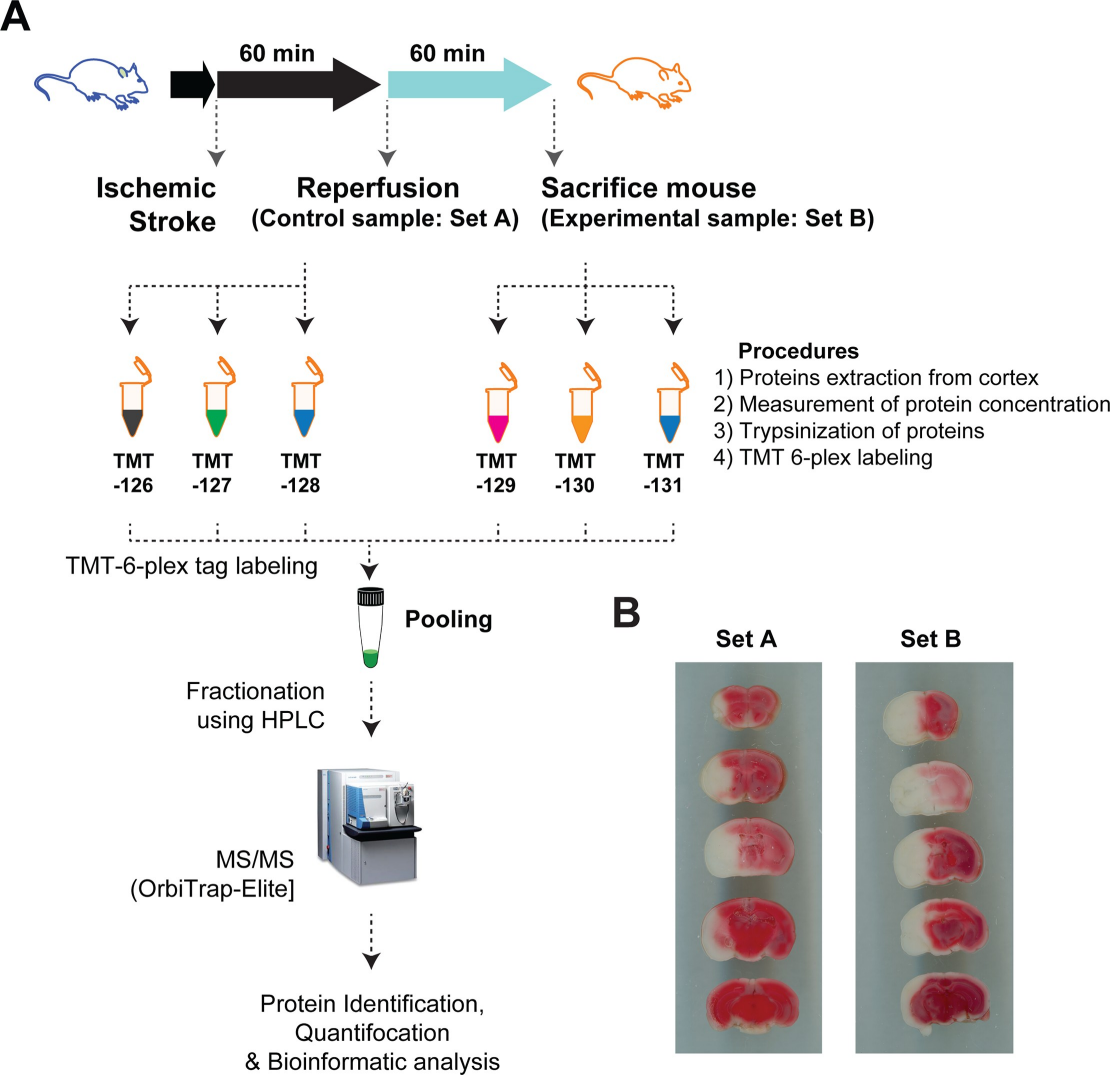
Experimental illustration (MCAO) and TTC-stained brain images post-reperfusion. (A) Experimental throughput, including mouse treatments, TMT-labeling, HPLC, and mass spectrometry are shown. (B) Images of sample mouse brain slices stained in TTC after 1-hour stroke (set A) and after 1-hour reperfusion (set B).

**Fig 2:**
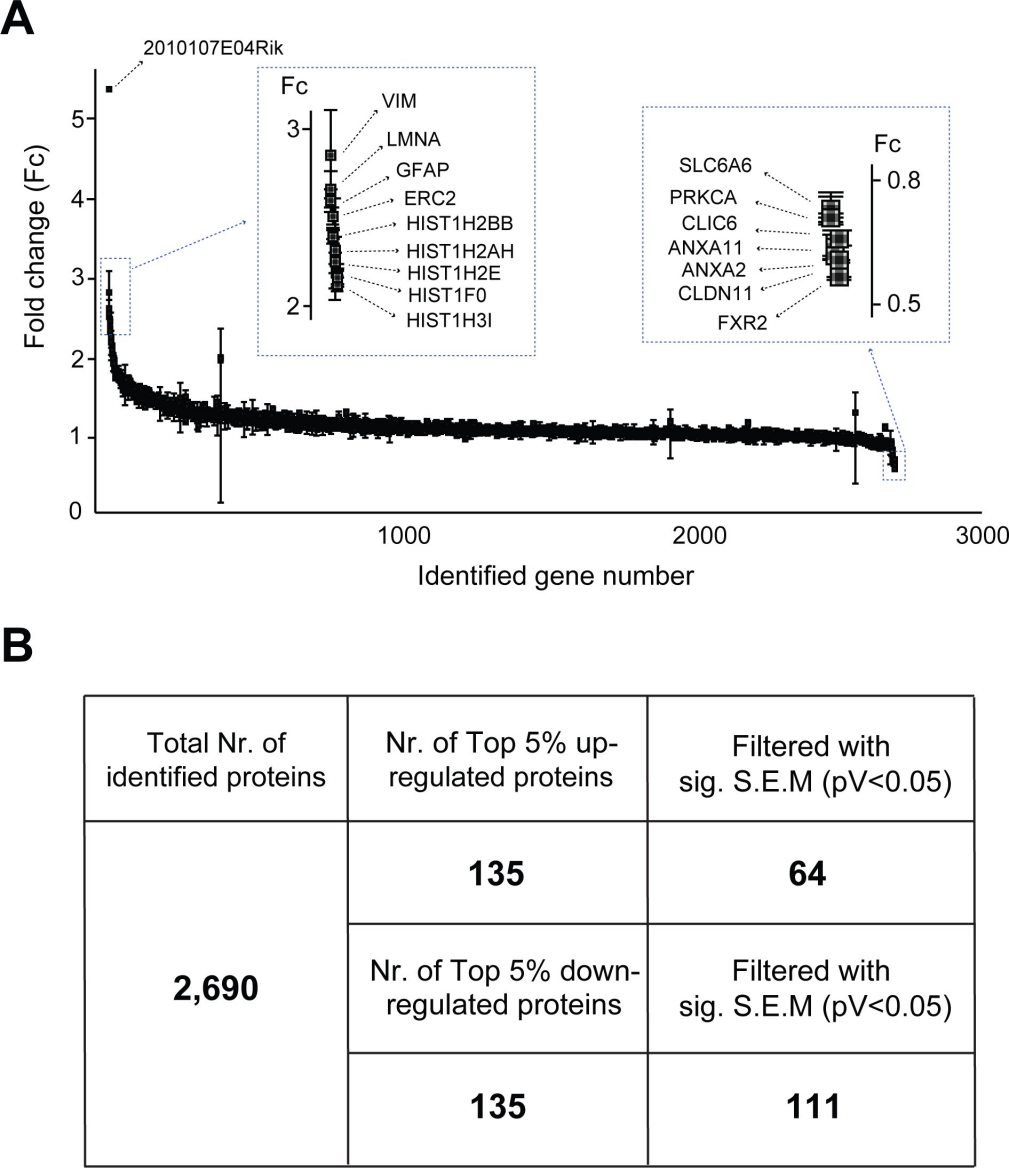
Protein quantification using TMT labeling. (A) Fold change of identified 2,690 proteins between post-stroke and post-reperfusion samples with standard deviation error bars. (B) Through TMT-labeling and mass spectrometry, 64 proteins had statistical significance among (top 5%) 135 up-regulated proteins, and 111 proteins had statistical significance among (top 5%) 135 down-regulated proteins.

This work represents a high-throughput analysis of proteins expressed during stroke and after 1 hour of recovery in order to identify large-scale families of protein-based molecular events in stroke pathology (Fig 1A). After performing MCAO on 6 male mice, half were sacrificed immediately after one hour of ischemia (set A in Fig 1B), and the remaining half were allowed to reperfuse for one hour before being sacrificed (set B in Fig 1B). Samples were TMT-labeled and fractionated via HPLC, then subsequently proteins were identified and quantified using a LTQ-Orbitrap Elite mass spectrometer interfaced with Easy-nLC II nanoflow LC system (Fig 1A). The identified 2,690 proteins were quantified using Proteome Discoverer v1.4 and represented in Fig 2A, S1 Table. A summary of up- and down-regulated protein counts is represented in Fig 2B.

### Functional alteration during 1-hour reperfusion in mouse cerebral cortex after ischemic stroke

**Fig 3:**
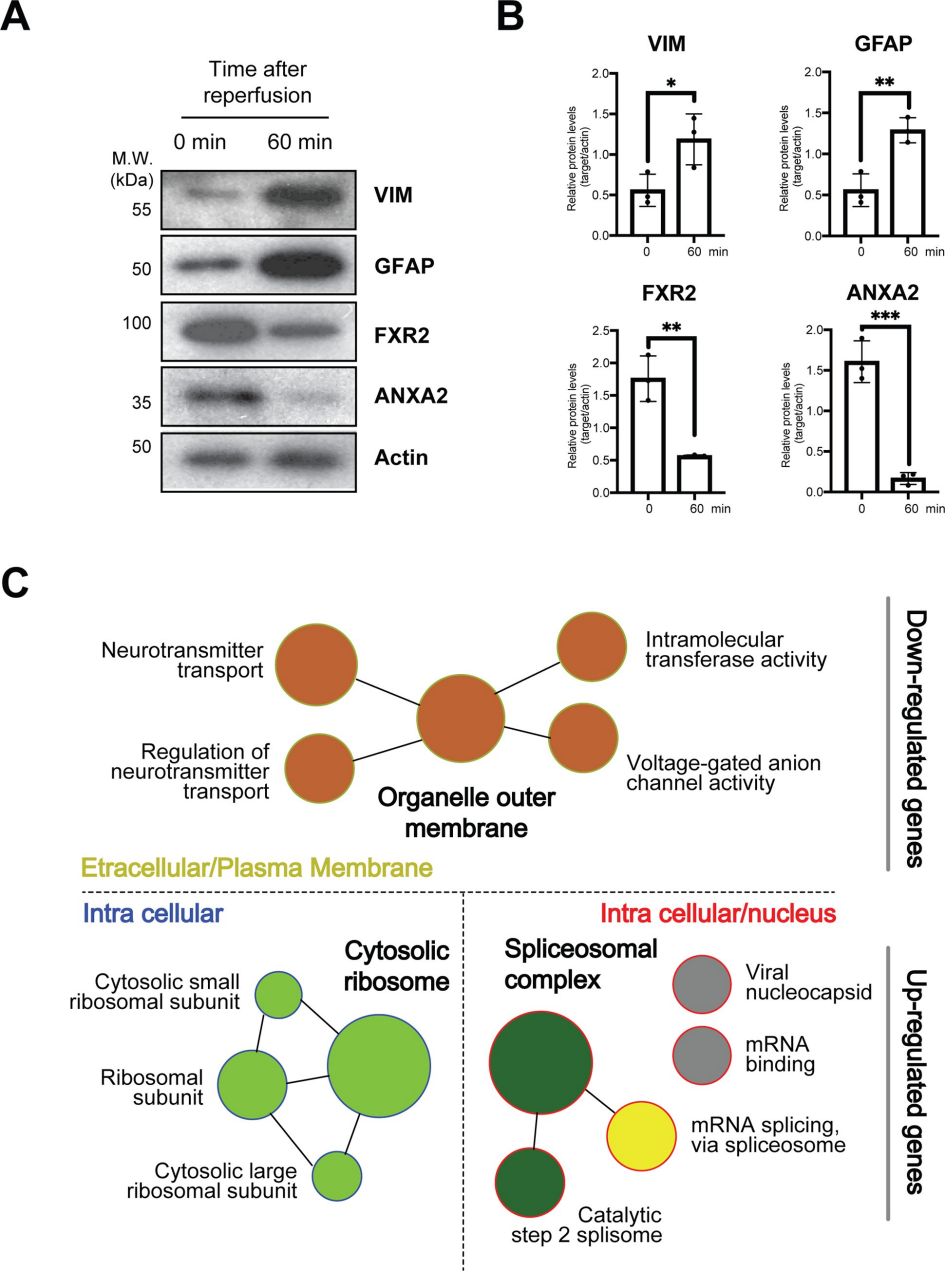
Network-based Gene Ontology (GO) analysis and functional domain frequencies. (A) Western blot validation of significantly up- and down-regulated proteins for Vimentin, Glial fibrillary acidic protein, Fragile X mental retardation syndrome-related protein 2, and Annexin a2. (B) Protein levels 0 and 60 min after reperfusion. (C) Network-based GO analysis yielded protein classification. Significantly down-regulated proteins (111 proteins) were mainly identified as organelle outer membrane proteins in the extracellular and plasma membrane regions whereas significantly up-regulated proteins (64 proteins) were found to be cytosolic ribosome proteins in the intracellular region or spliceosomal complex proteins in the intracellular and nucleus regions.

The four most significantly up- and down-regulated genes, Vimentin (VIM), Glial fibrillary acidic protein (GFAP), Fragile X mental retardation syndrome-related protein 2 (FXR2) and Annexin A2 (ANXA2), were validated using western blots and quantified (Fig 3A and 3B). In line with the mass spectrometry data, the western blots and quantification showed a significant increase in the amount of VIM and GFAP from 0 to 60 minutes post-reperfusion (p < 0.05 and p < 0.01, respectively). Similarly, FXR2 and ANXA2 showed significant decreases (p < 0.01 and p < 0.001, respectively). All significantly altered genes were further analyzed via network-based GO analysis and were found to have specific roles and locations depending on whether they were up- or down-regulated (Fig 3C, S2 Table).

### High similarity in distribution of protein domains and families between putative PAR-binding proteins and experimentally identified proteins in mouse cerebral cortex after ischemic stroke

**Fig 4:**
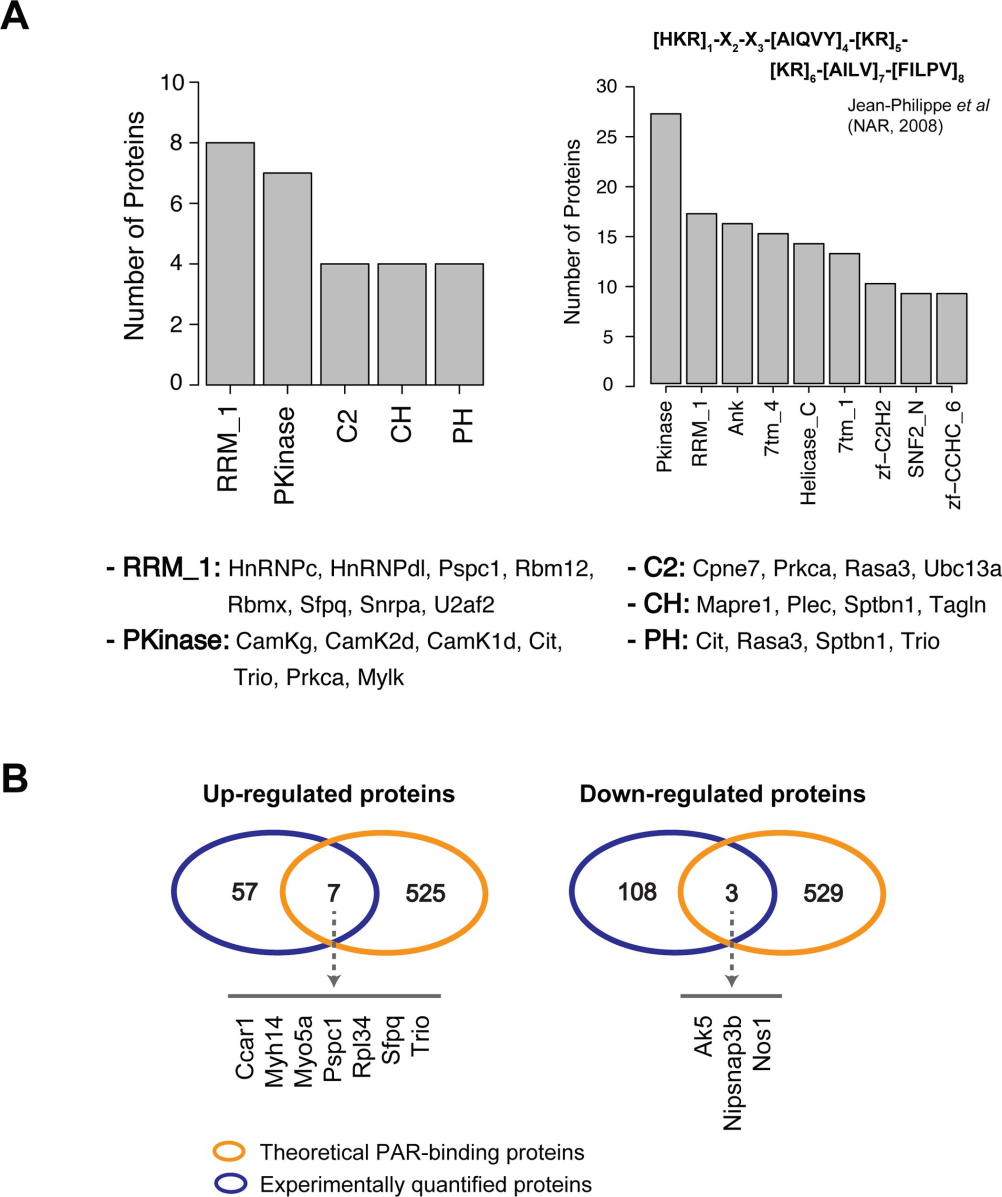
PAR-binding proteins. (A) Left: Proteins in the present study with the putative PAR-binding motif, categorized by main protein domains. Proteins in this chart are listed below. Right: The putative PAR-binding motif based on Jean-Philippe et al. (NAR, 2008) along with the numbers of proteins with the putative PAR-binding motif with specific domains. (B) The most significantly up- and down-regulated proteins were categorized by whether they are known to be experimentally proven PAR-binding proteins.

Distributions of protein domains and families were calculated and represented with the five functional domains and families that were most common amongst regulated proteins (Fig 4A, left). The RNA recognition motif (RRM) family, involved in translation-related processes, was the most populated with 8 identified proteins. Protein Kinase (PKinase) was the second most populated with 7 identified proteins. C2 and CH domains involved with zinc finger domains were identified with 4 proteins (Fig 4A, left). In collaboration with Gagné, J.P., et al., our group identified a putative PAR binding site (Fig 4A, right) and categorized identified proteins by functional domains and families [13]. In this study, all 64 of the top 5% of up-regulated proteins (p < 0.05) and all 111 of the top 5% of down-regulated proteins (p < 0.05) were checked for containing the PAR-binding motif and domains from the previous study. Both studies were highly enriched for protein kinase and RNA recognition motif domain-containing proteins. A*s* shown in Fig 4B, 7 up-regulated and 3 down-regulated proteins that were quantified in this study were identified as theoretical PAR-binding proteins.

## Discussion

Given the grand worldwide incidence of stroke and relative lack of understanding of stroke etiology, modern stroke studies have aimed to identify its underlying molecular mechanisms. Though previous studies have utilized high-throughput gene and protein expression experiments to understand the large variety of molecular events that occur during stroke and recovery, the present study used TMT-labeling, a far more comprehensive and accurate technique for protein detection and quantification (Figs 1 and 2). Furthermore, network-based gene ontology and clustering of protein domains were conducted to successfully identify families of molecular processes that may underlie stroke pathology (Fig 3C).

MCAO was performed on mice both after stroke and 1 hour of reperfusion (Fig 1B). The gene ontology and network analyses yielded several families of molecular functions which agreed with previous results: the spliceosome and synaptic transmission are involved in stroke pathology (Fig 3C) [3, 11]. The study also identified a cluster of cytosolic ribosome proteins implicated in stroke, as well as various functional domains shared amongst many up- or down-regulated proteins (Fig 3C). Because organelle outer membrane proteins were found to be down-regulated, it may be possible that cells attempted to limit intercellular communication post-stroke to prevent the spread of the effects of ischemia. Furthermore, cytosolic ribosome and spliceosomal complex proteins were found to be upregulated, indisputably showing translational alteration from stroke to reperfusion (Fig 3C and S1 Table).

Alongside Gagné, J.P., et al., our group identified cell death-related PAR-binding proteins, as well as a putative PAR-binding domain [13]. Briefly, cells were first damaged to trigger PAR-dependent cell death pathways, and then PAR antibodies were used to extract proteins before mass spectrometric analysis. Similarities were then identified between the PAR-binding proteins to create a putative PAR-binding site. In the present study, there was a high level of similarity between proteins significantly regulated after stroke recovery and involved in PAR-dependent cell death (Fig 4A and 4B). Amongst quantified proteins, the RNA recognition motif (RRM) and Protein Kinase (Pkinase) families were the most common, suggesting that translation after stroke is significantly impacted (Fig 4A). Furthermore, the C2, CH, and PH domains are all involved with cellular communication, potentially suggesting a role for these proteins in mitigating stroke damage to the penumbra and contralateral regions. Further studies may aim to perform knockouts of proteins containing these domains to observe potential differences in how far stroke damage travels to surrounding brain regions.

A comparison of significantly regulated proteins and known PAR-binding proteins revealed 7 up-regulated and 3 down-regulated PAR-binding proteins (Fig 4B). Overexpression of Ccar1 has been shown to lead to increased apoptosis [14], suggesting its role in cell death post-stroke. Sfpq, splicing factor proline and glutamine rich, in complex with NONO, may be required for non-homologous end joining (NHEJ) for DNA double-strand break repair [15]. Thus, upregulation of Sfpq may aid in DNA repair post-stroke. Trio, a guanine nucleotide exchange factor (GEF), limits dendrite formation in developing hippocampal neurons [16] and after dendrite formation, is involved in controlling synaptic function [16–18]. Furthermore, overexpression of a Trio isoform showed increased expression of AMPARs, suggesting a potential role for Trio in mediating neuronal communication post-stroke [19].

Amongst down-regulated proteins, Nos1 produces nitric oxide (NO) in the brain, a molecule associated with both neurotoxic and neuroprotective abilities in stroke [20, 21]. Though the true functional roles of these proteins have not been elucidated herein, future experiments may aim to explore their PAR-dependent roles in stroke and/or parthanatos.

Although the present study identified far more proteins than previous experiments, it only quantified 10% of the approximately 23,000 proteins in mice (Fig 2B). This may hail from limited capacity of mass spectrometry in both identification and quantification of entire mouse proteome in single MS/MS analysis. New technological advances may yield further protein identification and functional clusters.

Moreover, because the present study analyzed protein samples from the cerebral cortex, it would be prudent to repeat the experiments with brain dissection and region-by-region quantification in order to reveal which proteins are up- and down-regulated. This would afford an understanding of how different brain regions respond to stroke, based on their physiological function and distance from the stroke site. In addition, knockout and knockdown of proteins identified in this investigation would yield physiological roles for these proteins in stroke pathology and recovery. Finally, searching for other proteins that may not have been quantified or identified by TMT-labeling within identified domains would benefit the functional domain analysis.

## Supporting information

S1 Table List of identified genes with TMT quantification. (XLSX)

S2 Table Network-based GO analysis. (XLSX)

S1 Raw images (PDF)

S2 Raw images (PDF)

S3 Raw images (PDF)

S1 Data (XLSX)

S2 Data (TXT)
