# Supplementary figures and images for: Quantitative mass spectrometric analysis of the mouse cerebral cortex after ischemic stroke
Source: PLoS One. 2020 Apr 21;15(4):e0231978. doi: 10.1371/journal.pone.0231978 (PMC7173877; doi:10.1371/journal.pone.0231978)

Original blot images - Figure 3A

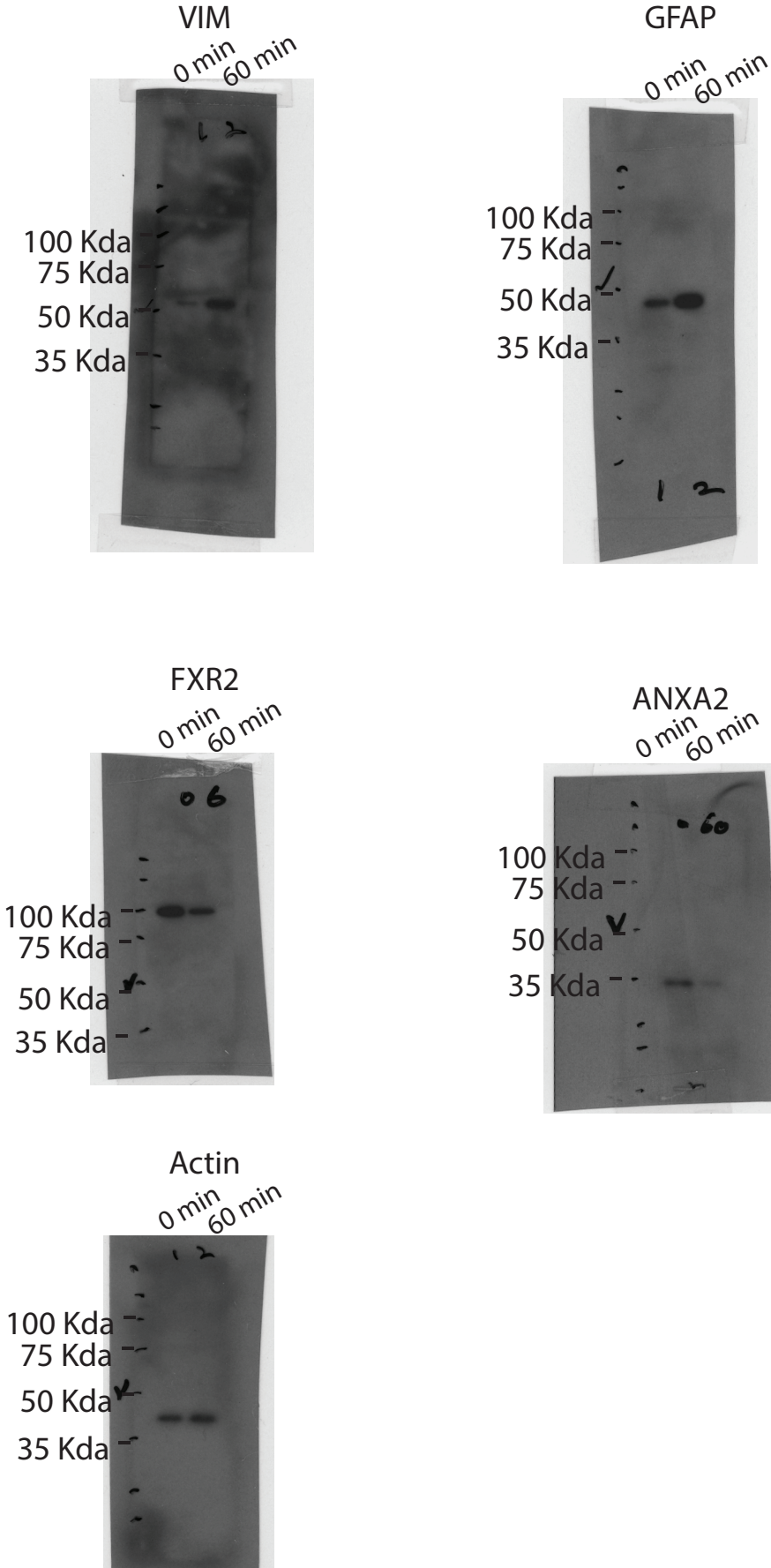

anti-vimentin.

Nov. 14. 2019. (1)

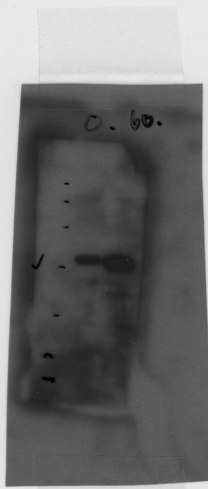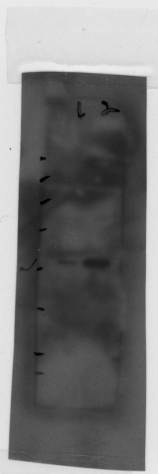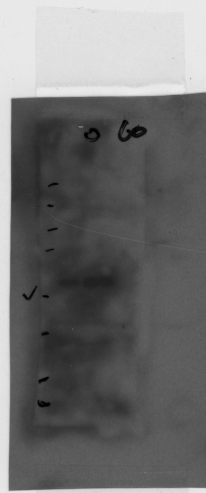

anti-FXR2.

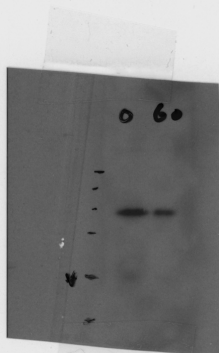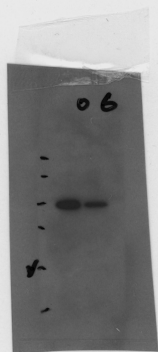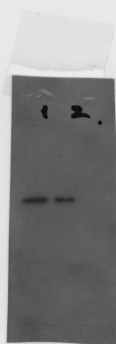

ent. - action -

Mar. 14. 2019. (2)

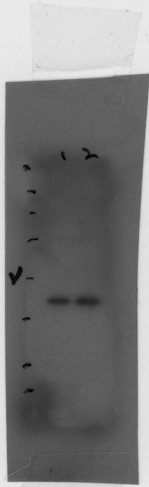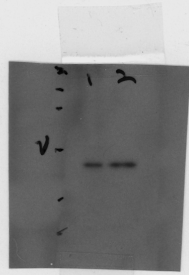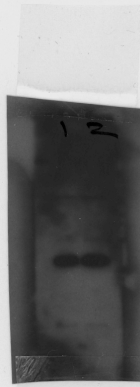

ent. -  
STAP.

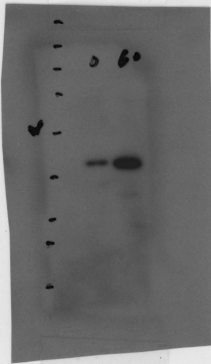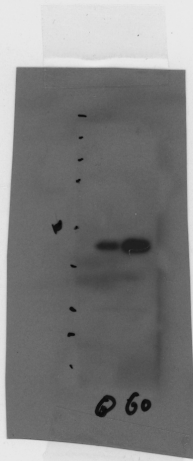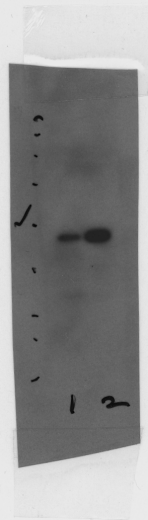

- annexin 2.

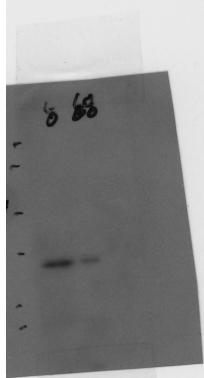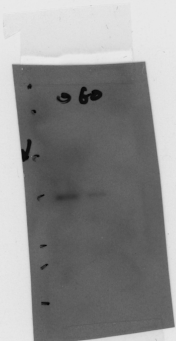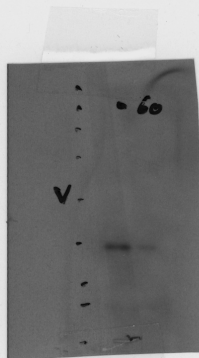

Supplement: S1 Raw images — (PDF) [file pone.0231978.s003.pdf]

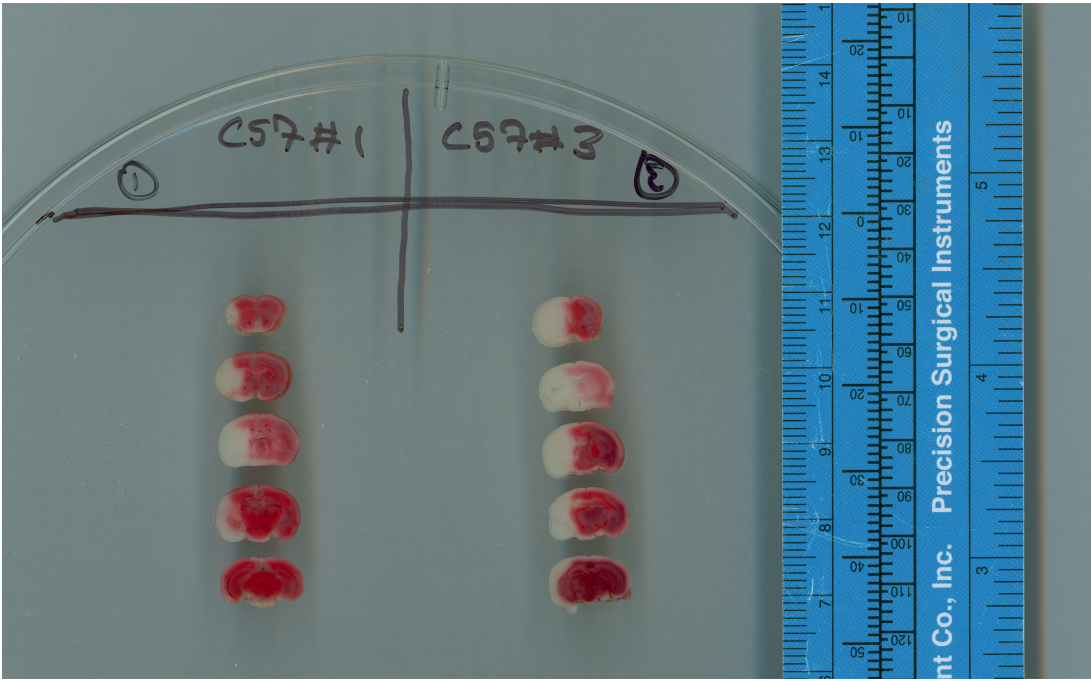

Supplement: S2 Raw images — (PDF) [file pone.0231978.s004.pdf]

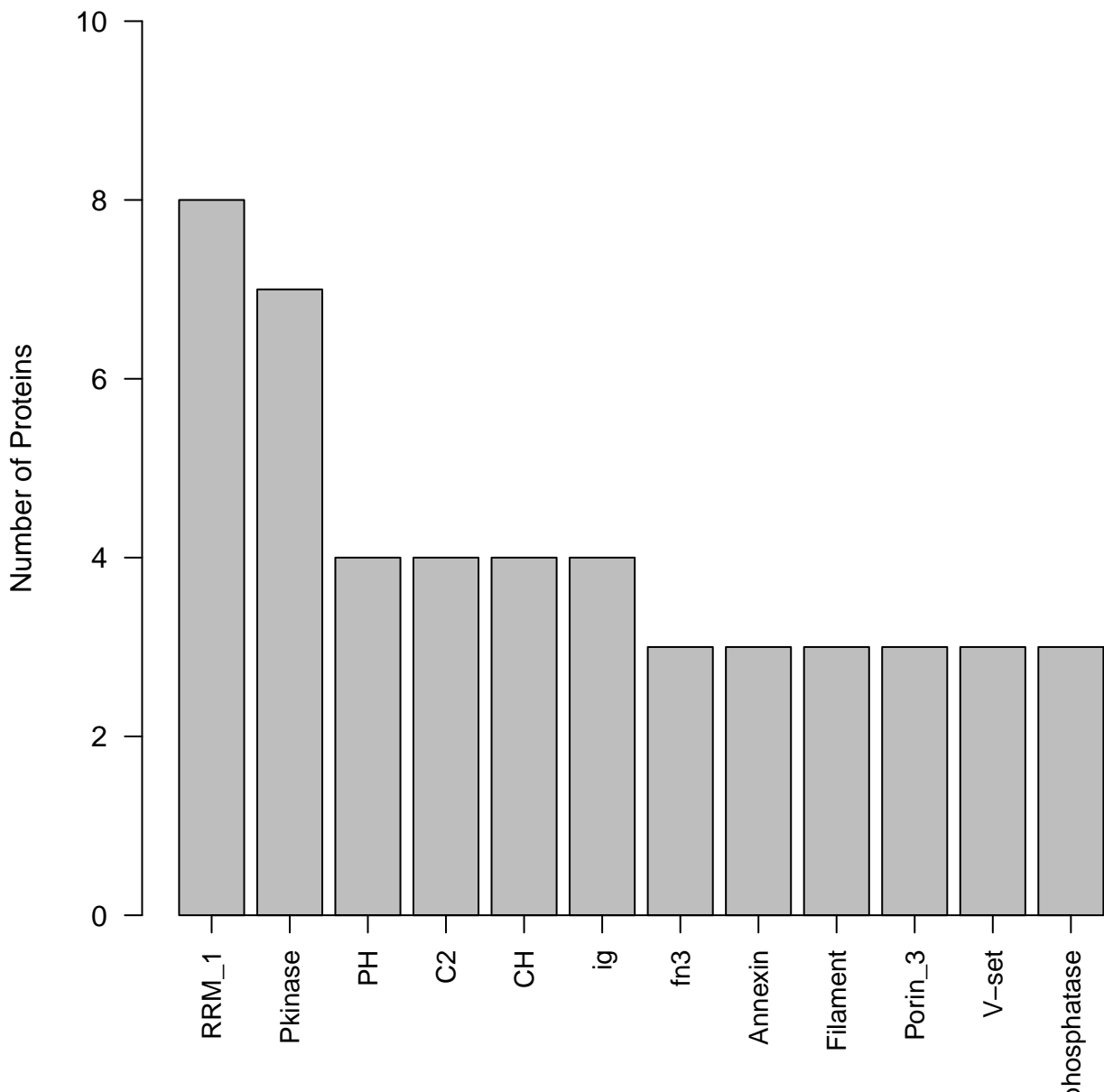

Supplement: S3 Raw images — (PDF) [file pone.0231978.s005.pdf]
